# 2-(4-Bromo­phen­yl)-2-oxoethyl anthracene-9-carboxyl­ate

**DOI:** 10.1107/S1600536812022684

**Published:** 2012-05-26

**Authors:** Hoong-Kun Fun, Safra Izuani Jama Asik, B. Garudachari, Arun M. Isloor, M. N Satyanarayan

**Affiliations:** a X-ray Crystallography Unit, School of Physics, Universiti Sains Malaysia, 11800 USM, Penang, Malaysia; b Organic Electronics Division, Department of Chemistry, National Institute of Technology–Karnataka, Surathkal, Mangalore 575 025, India; c Department of Physics, National Institute of Technology-Karnataka, Surathkal, Mangalore 575 025, India

## Abstract

In the title compound, C_23_H_15_BrO_3_, the anthracene ring system is essentially planar [maximum deviation = 0.29 (2) Å] and makes a dihedral angle of 5.74 (8)° with the mean plane of the bromo-substituted benzene ring. An intra­molecular C—H⋯O hydrogen bond generates an *S*(9) ring motif. In the crystal, mol­ecules are linked by C—H⋯O inter­actions, forming a two-dimensional network parallel to the *ac* plane. π–π stacking inter­actions are observed between benzene rings [centroid–centroid distances = 3.5949 (14) and 3.5960 (13) Å].

## Related literature

For background to the applications of anthracene, see: Bae *et al.* (2010 ▶); Reddy *et al.* (2011 ▶); Rather & Reid (1919 ▶). For hydrogen-bond motifs, see: Bernstein *et al.* (1995 ▶). For stability of the temperature controller used in the data collection, see: Cosier & Glazer (1986 ▶). 
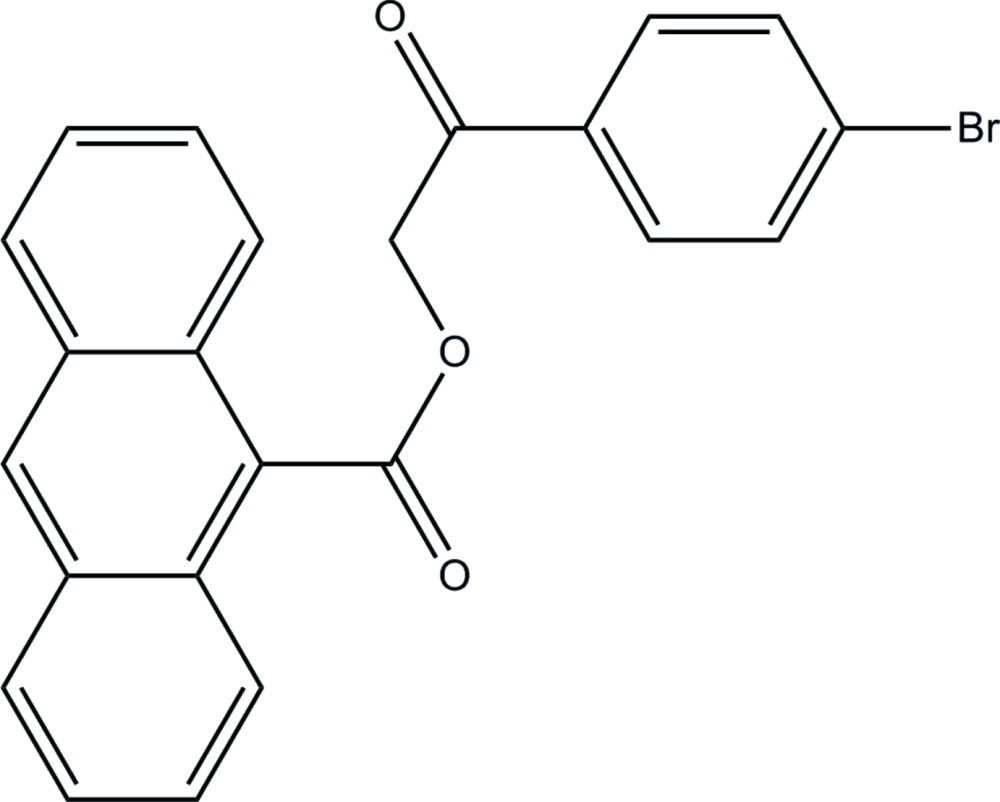


## Experimental

### 

#### Crystal data

C_23_H_15_BrO_3_
*M* *_r_* = 419.26
Monoclinic, 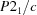

*a* = 10.1906 (8) Å
*b* = 15.0591 (12) Å
*c* = 13.7938 (8) Å
β = 122.376 (4)°
*V* = 1787.8 (2) Å^3^
*Z* = 4
Mo *K*α radiation
μ = 2.32 mm^−1^
*T* = 100 K
0.29 × 0.19 × 0.18 mm

#### Data collection

Bruker APEX DUO CCD area-detector diffractometer
Absorption correction: multi-scan (*SADABS*; Bruker, 2009 ▶) *T*_min_ = 0.550, *T*_max_ = 0.677
23513 measured reflections
6409 independent reflections
4600 reflections with *I* > 2σ(*I*)
*R*_int_ = 0.042

#### Refinement

*R*[*F* ^2^ > 2σ(*F* ^2^)] = 0.045
*wR*(*F* ^2^) = 0.141
*S* = 1.01
6409 reflections
244 parameters
H-atom parameters constrained
Δρ_max_ = 1.67 e Å^−3^
Δρ_min_ = −0.48 e Å^−3^

### 

Data collection: *APEX2* (Bruker, 2009 ▶); cell refinement: *SAINT* (Bruker, 2009 ▶); data reduction: *SAINT*; program(s) used to solve structure: *SHELXTL* (Sheldrick, 2008 ▶); program(s) used to refine structure: *SHELXTL*; molecular graphics: *SHELXTL*; software used to prepare material for publication: *SHELXTL* and *PLATON* (Spek, 2009 ▶).

## Supplementary Material

Crystal structure: contains datablock(s) global, I. DOI: 10.1107/S1600536812022684/rz2758sup1.cif

Structure factors: contains datablock(s) I. DOI: 10.1107/S1600536812022684/rz2758Isup2.hkl

Supplementary material file. DOI: 10.1107/S1600536812022684/rz2758Isup3.cml

Additional supplementary materials:  crystallographic information; 3D view; checkCIF report

## Figures and Tables

**Table 1:** Hydrogen-bond geometry (Å, °)

*D*—H⋯*A*	*D*—H	H⋯*A*	*D*⋯*A*	*D*—H⋯*A*
C12—H12*A*⋯O3	0.93	2.50	3.401 (3)	164
C20—H20*A*⋯O1^i^	0.93	2.47	3.394 (3)	176
C22—H22*A*⋯O3^ii^	0.93	2.31	3.199 (3)	159

Symmetry codes: (i) 
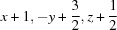
; (ii) 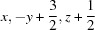.

**Fig. 1.**
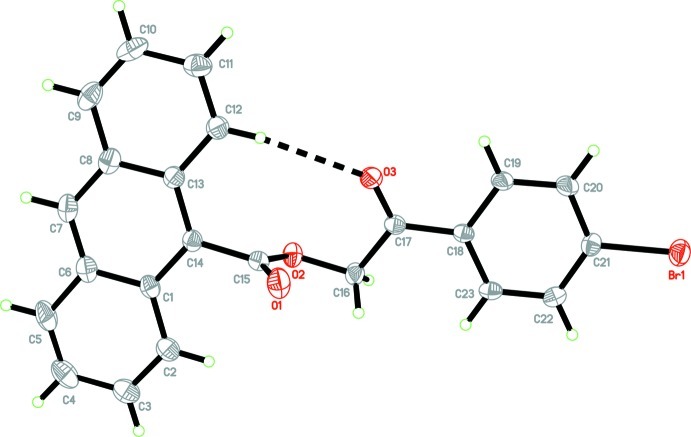
The structure of the title compound, showing 50% probability displacement ellipsoids. Hydrogen atoms are shown as spheres of arbitrary radius. An intramolecular hydrogen bond is shown as a dashed line.

**Fig. 2.**
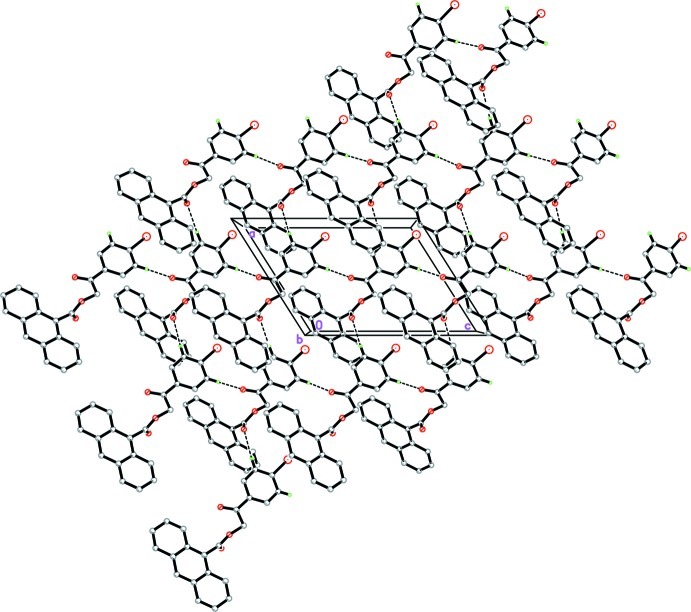
The crystal packing viewed along the b-axis, showing the molecules linked into a two-dimensional network parallel to ac plane. Hydrogen atoms not involved in hydrogen bonding (dashed lines) are omitted for clarity.

## supplementary crystallographic information

### Comment

Anthracene is a solid polycyclic aromatic hydrocarbon consisting of three fused benzene rings. It is used in the production of the dyes and organic semiconductor (Bae *et al.*, 2010) as high energy photons, electrons and alpha particle detectors and also used in blue light emitter in OLEDs (Reddy *et al.*, 2011) and identification of organic acids (Rather & Reid, 1919). Keeping this in view, the title compound (I) was synthesized to study its crystal structure.

In the title compound of (I), (Fig. 1), the anthracene (C1–C14) is essentially planar with maximum deviation of 0.029 (2) Å at atom C7 and makes a dihedral angle of 5.74 (8)° with the mean plane of bromo-substituted benzene (C18–C23) ring. The intramolecular C12—H12A···O3 interaction generates a S(9) ring motif (Bernstein *et al.*, 1995).

In the crystal structure of (Fig, 2), the molecules are linked by C20—H20A···O1 and C22—H22A···O3 interactions to form a two-dimensional network parallel to *ac* plane. π–π stacking interactions are observed between benzene rings with *Cg*2···*Cg*3 and *Cg*2···*Cg*4 distances of 3.5949 (14) and 3.5960 (13) Å, respectively. [*Cg*2 , *Cg*3 and *Cg*4 are the centroids of (C1/C6–C8/C13–C14), (C8–C13) and (C18–C23) rings, respectively.]

### Experimental

A mixture of anthracene-9-carboxylic acid (1.0 g, 0.0044 mol), potassium carbonate (0.589 g, 0.0043 mol) and 2-bromo-1-(4-bromophenyl)ethanone (0.746 g, 0.0053 mol) in dimethylformamide (10 ml) was stirred at room temperature for 1 h. On cooling, colourless needle-shaped crystals of 2-(4-bromophenyl)-2-oxoethyl anthracene-9-carboxylate separated. They were collected by filtration and recrystallized from ethanol. Yield: 1.60 g, 85.1 %, M.p. 421–423 K.

### Refinement

All the H atoms were positioned geometrically and refined using a riding model with C–H = 0.93 and 0.97 Å. The *U*_iso_ values were constrained to be 1.2*U*_eq_ of the carrier atom for the H atoms. Four outliers (-4 0 4), (0 2 1), (0 2 0) and (0 6 0) were omitted.

### Crystal data

**Table:**

C_23_H_15_BrO_3_	*F*(000) = 848
*M**_r_* = 419.26	*D*_x_ = 1.558 Mg m^−^^3^
Monoclinic, *P*2_1_/*c*	Mo *K*α radiation, λ = 0.71073 Å
Hall symbol: -P 2ybc	Cell parameters from 5144 reflections
*a* = 10.1906 (8) Å	θ = 2.5–31.7°
*b* = 15.0591 (12) Å	µ = 2.32 mm^−^^1^
*c* = 13.7938 (8) Å	*T* = 100 K
β = 122.376 (4)°	Block, colourless
*V* = 1787.8 (2) Å^3^	0.29 × 0.19 × 0.18 mm
*Z* = 4	

### Data collection

**Table:**

Bruker APEX DUO CCD area-detector diffractometer	6409 independent reflections
Radiation source: fine-focus sealed tube	4600 reflections with *I* > 2σ(*I*)
Graphite monochromator	*R*_int_ = 0.042
φ and ω scans	θ_max_ = 32.5°, θ_min_ = 2.5°
Absorption correction: multi-scan (*SADABS*; Bruker, 2009)	*h* = −15→15
*T*_min_ = 0.550, *T*_max_ = 0.677	*k* = −22→22
23513 measured reflections	*l* = −20→17

### Refinement

**Table:**

Refinement on *F*^2^	Primary atom site location: structure-invariant direct methods
Least-squares matrix: full	Secondary atom site location: difference Fourier map
*R*[*F*^2^ > 2σ(*F*^2^)] = 0.045	Hydrogen site location: inferred from neighbouring sites
*wR*(*F*^2^) = 0.141	H-atom parameters constrained
*S* = 1.01	*w* = 1/[σ^2^(*F*_o_^2^) + (0.0886*P*)^2^ + 0.0927*P*] where *P* = (*F*_o_^2^ + 2*F*_c_^2^)/3
6409 reflections	(Δ/σ)_max_ = 0.001
244 parameters	Δρ_max_ = 1.67 e Å^−^^3^
0 restraints	Δρ_min_ = −0.48 e Å^−^^3^

### Special details

**Table:**

Experimental. The crystal was placed in the cold stream of an Oxford Cryosystems Cobra open-flow nitrogen cryostat (Cosier & Glazer, 1986) operating at 100.0 (1) K.
Geometry. All esds (except the esd in the dihedral angle between two l.s. planes) are estimated using the full covariance matrix. The cell esds are taken into account individually in the estimation of esds in distances, angles and torsion angles; correlations between esds in cell parameters are only used when they are defined by crystal symmetry. An approximate (isotropic) treatment of cell esds is used for estimating esds involving l.s. planes.
Refinement. Refinement of F^2^ against ALL reflections. The weighted R-factor wR and goodness of fit S are based on F^2^, conventional R-factors R are based on F, with F set to zero for negative F^2^. The threshold expression of F^2^ > 2sigma(F^2^) is used only for calculating R-factors(gt) etc. and is not relevant to the choice of reflections for refinement. R-factors based on F^2^ are statistically about twice as large as those based on F, and R- factors based on ALL data will be even larger.

### Fractional atomic coordinates and isotropic or equivalent isotropic displacement parameters (Å^2^)

**Table:**

	*x*	*y*	*z*	*U*_iso_*/*U*_eq_	
Br1	0.87070 (3)	0.870236 (16)	0.94495 (2)	0.03087 (9)	
O1	0.1433 (2)	0.62950 (10)	0.30661 (16)	0.0325 (4)	
O2	0.29082 (18)	0.51653 (10)	0.41883 (13)	0.0240 (3)	
O3	0.50031 (19)	0.63375 (10)	0.44349 (14)	0.0261 (3)	
C1	−0.0111 (2)	0.44108 (13)	0.19350 (18)	0.0216 (4)	
C2	−0.0870 (3)	0.45257 (15)	0.2544 (2)	0.0260 (4)	
H2A	−0.0463	0.4923	0.3154	0.031*	
C3	−0.2193 (3)	0.40581 (17)	0.2243 (2)	0.0323 (5)	
H3A	−0.2672	0.4139	0.2651	0.039*	
C4	−0.2831 (3)	0.34522 (17)	0.1312 (3)	0.0371 (6)	
H4A	−0.3731	0.3140	0.1109	0.045*	
C5	−0.2135 (3)	0.33265 (15)	0.0721 (2)	0.0335 (5)	
H5A	−0.2562	0.2920	0.0120	0.040*	
C6	−0.0757 (3)	0.38009 (14)	0.0990 (2)	0.0257 (4)	
C7	−0.0046 (3)	0.36890 (14)	0.0382 (2)	0.0271 (4)	
H7A	−0.0492	0.3308	−0.0246	0.033*	
C8	0.1329 (2)	0.41350 (15)	0.06852 (18)	0.0253 (4)	
C9	0.2078 (3)	0.40118 (17)	0.0066 (2)	0.0308 (5)	
H9A	0.1644	0.3627	−0.0558	0.037*	
C10	0.3417 (3)	0.44494 (18)	0.0377 (2)	0.0338 (5)	
H10A	0.3901	0.4352	−0.0025	0.041*	
C11	0.4079 (3)	0.50561 (17)	0.1314 (2)	0.0307 (5)	
H11A	0.4992	0.5353	0.1519	0.037*	
C12	0.3393 (2)	0.52057 (15)	0.19129 (19)	0.0248 (4)	
H12A	0.3829	0.5612	0.2514	0.030*	
C13	0.1998 (2)	0.47415 (14)	0.16253 (17)	0.0218 (4)	
C14	0.1252 (2)	0.48732 (13)	0.22245 (17)	0.0200 (4)	
C15	0.1862 (2)	0.55352 (14)	0.31773 (18)	0.0212 (4)	
C16	0.3542 (3)	0.57382 (15)	0.51649 (18)	0.0240 (4)	
H16A	0.3999	0.5385	0.5860	0.029*	
H16B	0.2719	0.6096	0.5119	0.029*	
C17	0.4772 (2)	0.63405 (12)	0.52133 (18)	0.0195 (4)	
C18	0.5685 (2)	0.69144 (13)	0.62398 (17)	0.0183 (3)	
C19	0.7085 (2)	0.72715 (14)	0.64551 (17)	0.0225 (4)	
H19A	0.7410	0.7148	0.5955	0.027*	
C20	0.7993 (3)	0.78068 (14)	0.74042 (18)	0.0244 (4)	
H20A	0.8934	0.8035	0.7557	0.029*	
C21	0.7457 (2)	0.79908 (13)	0.81176 (18)	0.0215 (4)	
C22	0.6081 (2)	0.76560 (14)	0.79325 (18)	0.0227 (4)	
H22A	0.5750	0.7796	0.8425	0.027*	
C23	0.5203 (2)	0.71067 (14)	0.69956 (18)	0.0212 (4)	
H23A	0.4285	0.6863	0.6867	0.025*	

### Atomic displacement parameters (Å^2^)

**Table:**

	*U*^11^	*U*^22^	*U*^33^	*U*^12^	*U*^13^	*U*^23^
Br1	0.03828 (15)	0.03103 (13)	0.02549 (13)	−0.00894 (9)	0.01853 (11)	−0.00714 (8)
O1	0.0254 (8)	0.0250 (8)	0.0331 (9)	0.0049 (6)	0.0064 (7)	−0.0067 (6)
O2	0.0261 (8)	0.0231 (7)	0.0187 (7)	−0.0025 (6)	0.0094 (6)	−0.0007 (5)
O3	0.0233 (7)	0.0367 (9)	0.0196 (7)	−0.0046 (6)	0.0124 (6)	−0.0027 (6)
C1	0.0187 (9)	0.0187 (8)	0.0222 (10)	0.0003 (7)	0.0075 (8)	0.0013 (7)
C2	0.0236 (10)	0.0257 (10)	0.0273 (11)	−0.0005 (8)	0.0127 (9)	0.0023 (8)
C3	0.0282 (11)	0.0326 (12)	0.0380 (13)	−0.0033 (9)	0.0191 (11)	0.0031 (10)
C4	0.0275 (12)	0.0303 (11)	0.0485 (16)	−0.0099 (10)	0.0169 (12)	0.0008 (11)
C5	0.0266 (11)	0.0206 (10)	0.0389 (13)	−0.0058 (9)	0.0079 (10)	−0.0069 (9)
C6	0.0231 (10)	0.0191 (9)	0.0257 (10)	0.0033 (7)	0.0070 (9)	−0.0008 (7)
C7	0.0271 (11)	0.0216 (10)	0.0224 (10)	0.0016 (8)	0.0065 (9)	−0.0025 (8)
C8	0.0263 (11)	0.0223 (10)	0.0219 (10)	0.0089 (8)	0.0092 (9)	0.0002 (7)
C9	0.0365 (12)	0.0318 (11)	0.0199 (10)	0.0119 (10)	0.0123 (10)	0.0031 (9)
C10	0.0403 (13)	0.0424 (13)	0.0274 (11)	0.0161 (11)	0.0240 (11)	0.0090 (10)
C11	0.0256 (11)	0.0398 (12)	0.0286 (11)	0.0049 (9)	0.0157 (10)	0.0083 (10)
C12	0.0207 (9)	0.0302 (10)	0.0204 (10)	0.0029 (8)	0.0091 (8)	0.0006 (8)
C13	0.0185 (9)	0.0240 (9)	0.0195 (9)	0.0048 (7)	0.0079 (8)	0.0015 (7)
C14	0.0181 (9)	0.0200 (8)	0.0182 (9)	0.0022 (7)	0.0073 (7)	0.0000 (7)
C15	0.0191 (9)	0.0241 (9)	0.0220 (9)	−0.0012 (7)	0.0121 (8)	−0.0009 (7)
C16	0.0270 (10)	0.0276 (10)	0.0186 (9)	−0.0070 (8)	0.0131 (8)	−0.0035 (8)
C17	0.0180 (9)	0.0203 (9)	0.0203 (9)	0.0014 (7)	0.0104 (8)	0.0014 (7)
C18	0.0172 (8)	0.0199 (8)	0.0190 (9)	0.0009 (7)	0.0104 (7)	0.0027 (7)
C19	0.0222 (9)	0.0300 (10)	0.0196 (9)	−0.0043 (8)	0.0140 (8)	−0.0002 (8)
C20	0.0244 (10)	0.0290 (10)	0.0243 (10)	−0.0051 (8)	0.0160 (9)	−0.0003 (8)
C21	0.0265 (10)	0.0197 (9)	0.0184 (9)	−0.0007 (7)	0.0119 (8)	0.0003 (7)
C22	0.0253 (10)	0.0240 (9)	0.0226 (10)	0.0027 (8)	0.0154 (9)	0.0013 (8)
C23	0.0206 (9)	0.0252 (9)	0.0221 (9)	0.0012 (7)	0.0143 (8)	0.0031 (8)

### Geometric parameters (Å, º)

**Table:**

Br1—C21	1.908 (2)	C10—C11	1.424 (4)
O1—C15	1.205 (2)	C10—H10A	0.9300
O2—C15	1.342 (3)	C11—C12	1.356 (3)
O2—C16	1.430 (3)	C11—H11A	0.9300
O3—C17	1.216 (2)	C12—C13	1.436 (3)
C1—C14	1.406 (3)	C12—H12A	0.9300
C1—C2	1.425 (3)	C13—C14	1.405 (3)
C1—C6	1.434 (3)	C14—C15	1.494 (3)
C2—C3	1.373 (3)	C16—C17	1.520 (3)
C2—H2A	0.9300	C16—H16A	0.9700
C3—C4	1.418 (4)	C16—H16B	0.9700
C3—H3A	0.9300	C17—C18	1.485 (3)
C4—C5	1.350 (4)	C18—C19	1.400 (3)
C4—H4A	0.9300	C18—C23	1.400 (3)
C5—C6	1.436 (3)	C19—C20	1.387 (3)
C5—H5A	0.9300	C19—H19A	0.9300
C6—C7	1.382 (3)	C20—C21	1.385 (3)
C7—C8	1.401 (3)	C20—H20A	0.9300
C7—H7A	0.9300	C21—C22	1.380 (3)
C8—C13	1.426 (3)	C22—C23	1.384 (3)
C8—C9	1.430 (3)	C22—H22A	0.9300
C9—C10	1.361 (4)	C23—H23A	0.9300
C9—H9A	0.9300		
			
C15—O2—C16	115.81 (17)	C14—C13—C8	118.66 (19)
C14—C1—C2	122.51 (19)	C14—C13—C12	122.43 (19)
C14—C1—C6	118.6 (2)	C8—C13—C12	118.9 (2)
C2—C1—C6	118.9 (2)	C13—C14—C1	121.74 (19)
C3—C2—C1	121.0 (2)	C13—C14—C15	120.68 (18)
C3—C2—H2A	119.5	C1—C14—C15	117.56 (18)
C1—C2—H2A	119.5	O1—C15—O2	124.0 (2)
C2—C3—C4	120.3 (2)	O1—C15—C14	124.7 (2)
C2—C3—H3A	119.9	O2—C15—C14	111.23 (17)
C4—C3—H3A	119.9	O2—C16—C17	110.32 (16)
C5—C4—C3	120.2 (2)	O2—C16—H16A	109.6
C5—C4—H4A	119.9	C17—C16—H16A	109.6
C3—C4—H4A	119.9	O2—C16—H16B	109.6
C4—C5—C6	122.1 (2)	C17—C16—H16B	109.6
C4—C5—H5A	119.0	H16A—C16—H16B	108.1
C6—C5—H5A	119.0	O3—C17—C18	121.71 (18)
C7—C6—C1	119.7 (2)	O3—C17—C16	120.27 (18)
C7—C6—C5	122.7 (2)	C18—C17—C16	118.01 (17)
C1—C6—C5	117.6 (2)	C19—C18—C23	119.11 (19)
C6—C7—C8	121.7 (2)	C19—C18—C17	118.19 (17)
C6—C7—H7A	119.1	C23—C18—C17	122.70 (18)
C8—C7—H7A	119.1	C20—C19—C18	120.87 (19)
C7—C8—C13	119.6 (2)	C20—C19—H19A	119.6
C7—C8—C9	121.9 (2)	C18—C19—H19A	119.6
C13—C8—C9	118.6 (2)	C21—C20—C19	117.97 (19)
C10—C9—C8	121.0 (2)	C21—C20—H20A	121.0
C10—C9—H9A	119.5	C19—C20—H20A	121.0
C8—C9—H9A	119.5	C22—C21—C20	122.95 (19)
C9—C10—C11	120.3 (2)	C22—C21—Br1	118.15 (15)
C9—C10—H10A	119.9	C20—C21—Br1	118.86 (16)
C11—C10—H10A	119.9	C21—C22—C23	118.40 (18)
C12—C11—C10	120.8 (2)	C21—C22—H22A	120.8
C12—C11—H11A	119.6	C23—C22—H22A	120.8
C10—C11—H11A	119.6	C22—C23—C18	120.67 (19)
C11—C12—C13	120.5 (2)	C22—C23—H23A	119.7
C11—C12—H12A	119.7	C18—C23—H23A	119.7
C13—C12—H12A	119.7		
			
C14—C1—C2—C3	−179.5 (2)	C12—C13—C14—C15	2.1 (3)
C6—C1—C2—C3	0.5 (3)	C2—C1—C14—C13	179.01 (19)
C1—C2—C3—C4	−0.2 (4)	C6—C1—C14—C13	−1.0 (3)
C2—C3—C4—C5	0.5 (4)	C2—C1—C14—C15	−2.7 (3)
C3—C4—C5—C6	−0.9 (4)	C6—C1—C14—C15	177.28 (18)
C14—C1—C6—C7	−0.8 (3)	C16—O2—C15—O1	−1.3 (3)
C2—C1—C6—C7	179.3 (2)	C16—O2—C15—C14	−178.65 (16)
C14—C1—C6—C5	179.10 (19)	C13—C14—C15—O1	93.4 (3)
C2—C1—C6—C5	−0.9 (3)	C1—C14—C15—O1	−84.9 (3)
C4—C5—C6—C7	−179.0 (2)	C13—C14—C15—O2	−89.2 (2)
C4—C5—C6—C1	1.1 (4)	C1—C14—C15—O2	92.5 (2)
C1—C6—C7—C8	2.2 (3)	C15—O2—C16—C17	−78.0 (2)
C5—C6—C7—C8	−177.7 (2)	O2—C16—C17—O3	5.7 (3)
C6—C7—C8—C13	−1.9 (3)	O2—C16—C17—C18	−173.42 (17)
C6—C7—C8—C9	179.2 (2)	O3—C17—C18—C19	−17.1 (3)
C7—C8—C9—C10	−179.8 (2)	C16—C17—C18—C19	162.00 (19)
C13—C8—C9—C10	1.2 (3)	O3—C17—C18—C23	163.1 (2)
C8—C9—C10—C11	−1.4 (4)	C16—C17—C18—C23	−17.7 (3)
C9—C10—C11—C12	0.2 (4)	C23—C18—C19—C20	0.2 (3)
C10—C11—C12—C13	1.2 (3)	C17—C18—C19—C20	−179.53 (19)
C7—C8—C13—C14	0.1 (3)	C18—C19—C20—C21	−1.3 (3)
C9—C8—C13—C14	179.09 (19)	C19—C20—C21—C22	0.9 (3)
C7—C8—C13—C12	−178.89 (19)	C19—C20—C21—Br1	178.53 (16)
C9—C8—C13—C12	0.1 (3)	C20—C21—C22—C23	0.5 (3)
C11—C12—C13—C14	179.8 (2)	Br1—C21—C22—C23	−177.15 (15)
C11—C12—C13—C8	−1.3 (3)	C21—C22—C23—C18	−1.6 (3)
C8—C13—C14—C1	1.3 (3)	C19—C18—C23—C22	1.2 (3)
C12—C13—C14—C1	−179.75 (19)	C17—C18—C23—C22	−179.03 (18)
C8—C13—C14—C15	−176.91 (18)		

### Hydrogen-bond geometry (Å, º)

**Table:**

*D*—H···*A*	*D*—H	H···*A*	*D*···*A*	*D*—H···*A*
C12—H12*A*···O3	0.93	2.50	3.401 (3)	164
C20—H20*A*···O1^i^	0.93	2.47	3.394 (3)	176
C22—H22*A*···O3^ii^	0.93	2.31	3.199 (3)	159

Symmetry codes: (i) *x*+1, −*y*+3/2, *z*+1/2; (ii) *x*, −*y*+3/2, *z*+1/2.
